# Neurophysiological and BOLD signal uncoupling of giant somatosensory evoked potentials in progressive myoclonic epilepsy: a case-series study

**DOI:** 10.1038/srep44664

**Published:** 2017-03-15

**Authors:** Silvia F. Storti, Alessandra Del Felice, Laura Canafoglia, Emanuela Formaggio, Francesco Brigo, Franco Alessandrini, Luigi G. Bongiovanni, Gloria Menegaz, Paolo Manganotti

**Affiliations:** 1 Department of Computer Science, University of Verona, Verona, Italy; 2 Department of Neuroscience-DSN, University of Padova, Padova, Italy; 3 Neurophysiopathology and Epilepsy Centre, IRCCS Foundation Carlo Besta Neurological Institute, Milan, Italy; 4 Foundation IRCCS San Camillo Hospital, Venice, Italy; 5 Franz Tappeiner Hospital, Department of Neurology, Merano, Italy; 6 University Hospital Verona, Department of Neuroradiology, Verona, Italy; 7 Department of Neurosciences, Biomedicine and Movement Sciences, University of Verona, Italy; 8 Department of Medical, Surgical and Health Sciences, Clinical Neurology Unit, Cattinara University Hospital, Trieste, Italy

## Abstract

In progressive myoclonic epilepsy (PME), a rare epileptic syndrome caused by a variety of genetic disorders, the combination of peripheral stimulation and functional magnetic resonance imaging (fMRI) can shed light on the mechanisms underlying cortical dysfunction. The aim of the study is to investigate sensorimotor network modifications in PME by assessing the relationship between neurophysiological findings and blood oxygen level dependent (BOLD) activation. Somatosensory-evoked potential (SSEP) obtained briefly before fMRI and BOLD activation during median-nerve electrical stimulation were recorded in four subjects with typical PME phenotype and compared with normative data. Giant scalp SSEPs with enlarger N20-P25 complex compared to normal data (mean amplitude of 26.2 ± 8.2 μV after right stimulation and 27.9 ± 3.7 μV after left stimulation) were detected. Statistical group analysis showed a reduced BOLD activation in response to median nerve stimulation in PMEs compared to controls over the sensorimotor (SM) areas and an increased response over subcortical regions (*p* < *0.01, Z* > *2.3,* corrected). PMEs show dissociation between neurophysiological and BOLD findings of SSEPs (giant SSEP with reduced BOLD activation over SM). A direct pathway connecting a highly restricted area of the somatosensory cortex with the thalamus can be hypothesized to support the higher excitability of these areas.

Progressive myoclonic epilepsies (PMEs) are a group of rare genetic disorders with geographical and ethnic variations, characterized by worsening myoclonus, generalized seizures, and progressive neurological deterioration including cerebellar dysfunction and dementia. PMEs may affect all ages, but typically present in late childhood or adolescence. The prognosis is generally poor, with people with PME eventually wheel-chair bound and with reduced life expectancy^1^.

PMEs differ from juvenile myoclonic epilepsy on the following aspects: i) complex phenotype including epilepsy plus movement disorder (action myoclonus); ii) progressive neurological disability; iii) failure to respond to antiepileptic drugs; iv) slowing of background electroencephalographic (EEG) activity^2^; v) presence of giant evoked potentials^3^.

The PMEs core phenotype results from different diseases that have heterogeneous genetic backgrounds, the most frequent being Unverricht-Lundborg disease (ULD), Lafora disease (LD), and other rarer pathologies^4^.

In PMEs, myoclonus has a cortical correlate disclosed by the analysis of EEG-electromyography (EMG) coupling, with a time-locking of myoclonic muscle contraction and spikes on EEG. Other signs of cortical hyper-excitability include somatosensory evoked potentials (SSEPs) of increased amplitude, known as giant-evoked potentials.

The enlarged amplitude of SSEPs, strongly suggestive of an increased excitability in response to incoming stimuli, allows to investigate how stimuli are processed, and which is the balance between excitatory and inhibitory phenomena^5^,^6^,^7^,^8^.

Functional magnetic resonance imaging (fMRI) is a non-invasive technique that measures hemodynamic changes associated with neuronal activation in the brain using blood oxygen level dependent (BOLD) contrast. The BOLD signal does not directly reflect neuronal activity, but arises from changes in hemodynamic properties. It consists of several contributions: the neuronal response to a stimulus, the complex relationship between neuronal activity and its hemodynamic response, the hemodynamic response itself, and how the MRI scanner detects it^9^. The neurovascular coupling between the neuronal activity and hemodynamic response has been largely discussed^10^ and models that characterize dynamics and features of the hemodynamic responses evoked by a neural activity have been suggested^11^. In addition, there has been growing interest in studying the potential complexity of the relationship between fMRI and simultaneous electrophysiological measurements, such as EEG^12^ or SSEPs^13^,^14^.

In PME subjects, motor tasks inside the MRI scanner may not be feasible due to the large motion artefacts. A passive stimulus, such as an electrical peripheral stimulation, combined with fMRI would contribute to assess the cortical function. BOLD response to median-nerve stimulation in controls has been widely described^15^,^16^. Seminal studies compared the evoked EEG potential and the fMRI response to somatosensory stimulation in normal subjects reporting a parallel increase of SSEP amplitude and BOLD signal with increasing stimulus intensity. This finding has been interpreted as strongly suggestive of the linear neurovascular coupling relationship^9^,^13^,^17^. In healthy volunteers, increasing the stimulus frequency decreases SSEP amplitude. The most likely explanation is that the complex inhibitory mechanisms mediated by gamma-aminobutyric acid (GABAergic) connections within the parietal cortex reduce the excitatory postsynaptic potential on those cells generating the SSEP components. Conversely, the BOLD increase could reflect the intensification of inhibitory circuits that produce the reduction of SSEP components^18^.

The pathogenesis of myoclonus in PME relies on abnormal processing of sensory input, with the presence of giant SSEPs, typically associated with increased long-latency reflexes, and hyper-excitable motor responses to afferent stimulation^18^. In a preliminary study, based on the enlarged topographical diffusion of the SSEP cortical components recorded in one subject with PME, we hypothesized an augmented, widespread BOLD signal over multiple cortical areas, especially in the parietal and frontal regions. Contrary to our expectations, giant SSEPs and a highly focal BOLD activation of the contralateral sensorimotor areas during median-nerve electrical stimulation were detected^19^.

The aim of this study is to confirm the initial observation of dissociation between neurophysiological findings and BOLD activation in a case series with definite PME, and identify possible sensorimotor network modifications in PME.

## Results

Four subjects with PME (28–52 years; mean 37.5 years) were enrolled. Three participants (nos 1, 2, and 4) had genetically confirmed diagnosis of ULD: cystatin B (CSTB) gene mutation in homozygosis (nos 1 and 2) or compound heterozygosis (no. 4).

Subject no. 3 was diagnosed with a *KCNC1* mutation^20^. The phenotype was consistent: onset of the symptoms was between 6 and 16 years; at the moment of our observation only the youngest subjects still showed recurrent seizures (nos 1 and 3), while all of the participants had mild to severe myoclonus resistant to multiple treatments. EEG showed epileptic discharges in three subjects (nos 1, 3, and 4) (see Table 1, “EEG discharges”).

**Figure 1:**
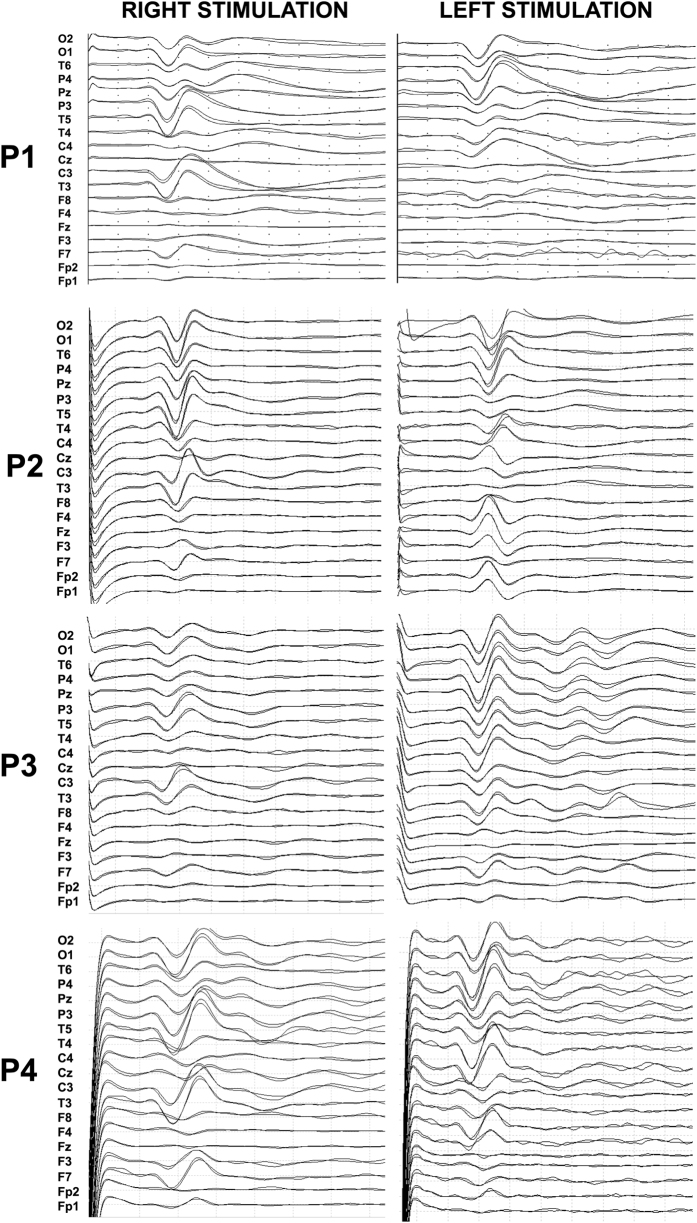
Diffuse high-amplitude SSEPs obtained after right (**A**) and left (**B**) median-nerve electrical stimulation at 3 Hz (scale: 20μV/div; time window: 100 ms).

Averaged SSEPs of PME subjects following right and left median nerve stimulation outside the scanner are displayed in Fig. 1.

All participants presented high-amplitude N20-P25 SSEP cortical components with widespread diffusion over the parietal regions.

After median-nerve electrical stimulation at 3 Hz, N20 showed a latency of 21.41 ± 1.4 ms (right) and 21.7 ± 2.0 ms (left), P25 showed a latency of 26.7 ± 1.6 ms (right) and 26.2 ± 2.2 ms (left), and a peak-to-peak N20-P25 amplitude of 26.2 ± 8.2 μV (right) and 27.9 ± 3.7 μV (left).

PME subjects had significantly larger N20-P25 peak amplitude compared to normative literature data^21^,^22^ (*p* < 0.001) [Reference values for the amplitude of the N20-P25 complex reported in Cruccu *et al*.^21^: 3.2 μV  ± 0.8; and in Canafoglia *et al*.^22^: 4.3 μV ± 2.7].

**Figure 2:**
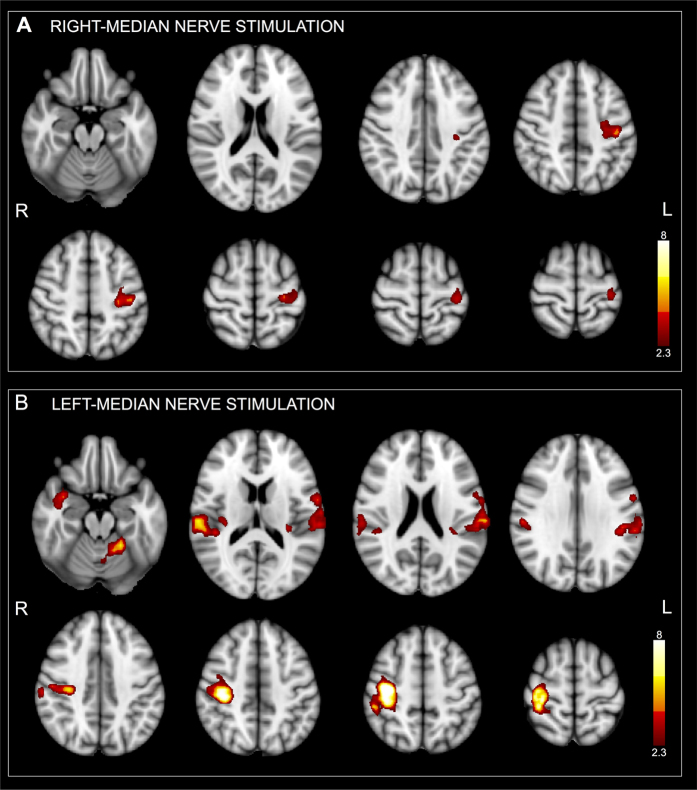
Fixed-effect group analysis for PME subjects during right (**A**) and left (**B**) median-nerve electrical stimulation at 3 Hz overlaid on 3D anatomical images in the MNI space. The color bar on the right indicates the statistical *Z* scores. Maps are thresholded by *Z* > *2.3* and a significance of *p* < *0.01* (FWE-corrected). Images are displayed in radiological convention.

Four right-handed healthy subjects matched for age (34–40 years; mean 41 years) were also enrolled as a control group for the neuroimaging part of the experiment. During right median-nerve electrical stimulation inside the scanner, fMRI group analysis of PME subjects showed a focal activity in the contralateral precentral and postcentral gyrus (PRG.L, POG.L) (Fig. 2A, Table 2). During the left median-nerve stimulation, group analysis showed the involvement of the contralateral PRG.R, POG.R, PO.R, Heschls gyrus (H.R) and insular cortex (INS.R), ipsilateral central opercular cortex (CO.L), POG.L and INS.L, and ipsilateral cerebellum (V) (Fig. 2B, Table 2). Although the group analysis showed smaller activation clusters during the right median-nerve stimulation than during the left stimulation, the areas had a high degree of co-localization. Activation maps were thresholded at *Z* > *2.3, p* < *0.01*, family-wise error (FWE)-corrected.

**Figure 3:**
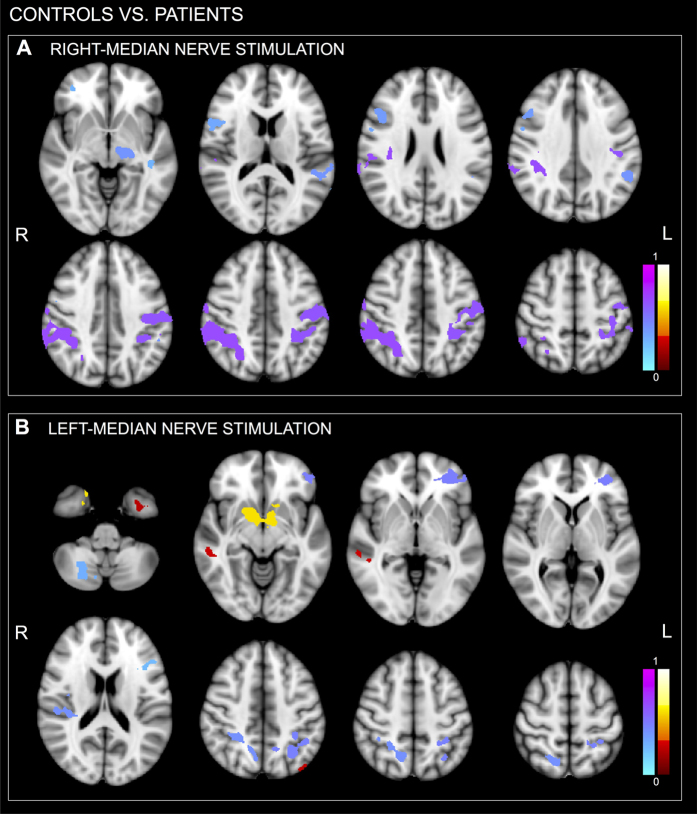
Controls vs. patients statistical group results assessed using a non-parametric method with a significance threshold of *p* < 0.01, FWE-corrected cluster extent threshold pFWE < 0.05. The maps show for both right (**A**) and left (**B**) median-nerve electrical stimulations the difference *controls-patients*. Cool colour represents areas more prominently activated in controls than in patients, whereas hot color represents areas more prominently activated in patients than in controls. Images are displayed in radiological convention.

Statistical group analysis showed a reduced BOLD activation in the PME group compared to the control group, in particular over the sensorimotor areas. The resulting cluster *p*-values are displayed in Fig. 3 and given in Table 3 (cluster defining threshold of *p* < *0.01, Z* > *2.3*, only clusters consisting of more than 1000 voxels are displayed). Motor areas (e.g. POG, CER, and CO) were more strongly activated in controls than in patients.

## Discussion

PMEs displayed a highly focal BOLD SSEP-related activation over sensorimotor areas. The extent of cortical areas activated in PME subjects was overall reduced compared to controls.

PMEs are characterized by an enhanced cortical excitability, associated with high amplitude positive potential following N20 cortical potential epitomized by giant SSEPs. A hypothesis is that a defective post-excitatory inhibition could contribute to the phenomenon^6^,^7^. Unclear appears the cascade through which they are generated. Epidural SSEP recordings provided evidence of a possible involvement of the primary somatosensory cortex (SI) and the primary motor cortex (MI), with an excitatory wave travelling from the SI to MI^23^.

A contrasting view on giant SSEPs in ULD is that of the existence of a subcortical loop, that “short-cuts” cortical areas. Nonetheless, this hypothesis does not take into account the EEG-EMG time locking of myoclonus that suggests instead a cortical generator. Alternatively, a direct pathway connecting the thalamus with SI would support the higher excitability of these areas^22^. The existence of this preferential connection, postulated on the basis of cortical relay conduction time, would support our finding of a highly restricted BOLD activation area to which sensory stimuli would be projected on the cortex, skipping the physiological processing of the incoming stimulus over multiple cortical areas. The existence of a preferential connection between thalamus and SI could support our findings: the incoming sensory stimulus would be directly channelled to SI, reducing both relée time and polysynaptic activations. The observed neuronal discharge would thus take place mainly over the cortex directly targeted by the thalamus – SI in the specific case.

This short-cut would also explain the extreme impoverishment of the physiological sensorimotor network. In healthy subjects a median nerve non-painful stimulus activates the SI, bilateral secondary somatosensory area (SII) and bilateral insula^18^. Conversely, in PME subjects ipsilateral activations were extremely reduced or missing, stressing the highly selective involvement of the contralateral SI.

Another interpretation of the results rests on the BOLD signal characteristics. The highly focal BOLD activation restricted to the contralateral sensorimotor areas in subjects with PME is intriguing, based on the possible dissociation between neurophysiological and BOLD data (i.e. giant SSEP and focal BOLD), and could highlight the fact that the BOLD signal can potentially provide information that is complementary to that provided by neurophysiology.

An enlarged SSEP could intuitively be expected to be generated by a larger cortical area, or even network, than that activated in physiological conditions. Instead, a restricted area of activation and an almost missing neuronal network were detected. An explanatory mechanism rests on the not yet established nature of the BOLD response. The underlying effect generating BOLD is determined by the paramagnetic properties of deoxyhemoblogin, which distorts the magnetic field. Indeed, it also depends strongly on the coupling ratio of the fractional changes in cerebral blood flow (CBF) and cerebral metabolic rate of oxygen (CMRO_2_)^24^. This ratio tracks the inhibitory to excitatory activity in the neural response. A strong inhibitory activity is usually related to a greater CBF increase compared to CMRO_2_. Unfortunately, the definition of this ratio is not yet part of the standard fMRI assessment: we cannot thus determine by which activity the BOLD signal we record after a SSEP is generated.

It is also possible that the very focal and restricted activity over the SI derives from the balance between a strongly decreased GABAergic inhibitory network activity and the consequently increased neuronal discharge of the involved SI. In addition, the poor temporal resolution of fMRI hampers the attempt to determine to which degree the evoked cortical components correlate with BOLD activation. Experimental studies suggest, however, a strong correlation between hemodynamic response and neuronal activity, although the former tends to be spatially more widespread and longer lasting than the latter. Combining multiple techniques performed either simultaneously or in separate sessions may therefore be useful to overcome the limitations of spatial and temporal resolution^25^.

All the recruited subjects were on antiepileptic drugs, including GABAergic compounds. We know that N24/P24 and P22 SSEP components are probably generated by deep spiny cell hyperpolarization, which is strongly increased by inhibitory inputs from GABAergic interneurons. There is thus a clear influence of inhibitory circuitry in shaping these SSEP components^26^,^27^,^28^. In fact, the effect of antiepileptic drugs on SSEP is a very poorly documented area, with the scant previous data either negative or non-informative; the few data reported in literature^27^ on the modulatory effect of GABAergic drugs show an increase of SSEP amplitudes in healthy volunteers that is nonetheless negligible if compared with the giant SSEP amplitudes of PME.

A possible dissociation between neurophysiological and BOLD responses is also supported by experiments based on the simultaneous EEG-fMRI and EEG-time domain functional near-infrared spectroscopy (TD-fNIRS) recordings in subjects with PME^29^. EEG-fMRI showed characteristic changes in movement associated EEG event-related desynchronization/synchronization (ERD/ERS) in alpha and beta bands in PME, with an increase of desynchronization in the alpha band and an absent or extremely reduced beta rebound at movement end^29^. The desynchronization increase in the alpha band is interpreted as a correlate of pre-activation during planning of the movement, and suggests increased cortical excitation. Conversely, the absent post-movement synchronization in the beta band suggests the decrease of inhibitory GABAergic neurons^30^. Of note, no difference in fMRI features related to this ERD/ERS activity was detected. This dissociation can be explained by the temporal dynamic of activity of EEG (with its optimal sensitivity to temporal resolution) and fMRI (with its low temporal resolution), and supports our hypothesis of an imbalance between the excitatory and inhibitory components, and the related CBF and CMRO_2_. Additional evidence supporting this hypothesis is provided by EEG/fMRI and fNIRS recordings in ULD subjects compared to controls^31^. The fMRI and TD-NIRS resulted significantly correlated, and showed smaller hemodynamic changes in the patients group. A possible explanation is that the loss of inhibitory neurons, typically firing at very high frequencies, not only causes hyperexcitability and impaired beta ERS, but also reduces the metabolic requirement, as reflected by lower hemodynamic curves measured both by TD-fNIRS and BOLD.

In conclusion, we observed highly focal BOLD activation in the sensorimotor areas in PME persons with giant-evoked potentials, suggesting focal sensorimotor cortex hyperexcitability in the absence of epileptiform abnormalities. Further research into the cortical genesis of giant SSEPs in subjects with PMEs needed.

## Methods

### Participants

Four subjects with a typical PME phenotype were recruited (Table 1). Amplitudes of cortical SEEP components measured outside the scanner were compared to reference values from normative published data^21^,^22^. Four right-handed healthy subjects served as a control group for the fMRI session. The subjects provided written informed consent. The study was approved by the Local Ethics Committee of the University Department and Hospital of Verona, and conducted in accordance with the Declaration of Helsinki.

### SSEPs

Cortical SSEP recordings, performed outside the scanner, were obtained by stimulating the right and left median nerve (square-wave stimuli, 0.1 ms, 3 Hz). EEG data were acquired at a sampling rate of 512 Hz using a cabled cap (SEI EMG s.r.l, Padua, Italy) with 21 electrodes (reference anterior to Fz and ground posterior to Pz) positioned according to the international 10–20 system of electrode and recorded using a Micromed System (Micromed, Treviso, Italy). Impedance was kept below 10 kΩ. To locate the right and left median-nerve at the wrist, the stimulation intensity and electrode sites were modified until stimulation produced an observable thumb twitch. The necessary current for reaching the motor threshold varied between patients (range 8–24 mA) and was kept constant for each.

Averaged potentials were obtained from 500 artifact-free responses. All tests were repeated at least twice to ensure reliability of responses^19^. Among the cortical potentials, peak latencies and amplitudes of the N20 and P25 potentials were collected, as these components are likely to be generated in the somatosensory cortex. The N20-P25 amplitudes of PME subjects were statistically compared with those recorded in a group of 18 healthy subjects (11 women, 7 men; mean age 35.3 ± 12.2 years)^22^ and with those recorded in a young population^21^ using a two-tailed *z*-test (*p* < 0.001).

### MRI data acquisition and experimental paradigm

Inside the bore of the scanner participants laid supine on a bed, with elbows flexed at 120° and hands pronated in a relaxed position, and the head restrained with adjustable pads on both sides. They were instructed to lie still inside the scanner with their eyes closed and not to fall asleep. All participants wore earplugs.

MRI data were acquired on a 3 T MR scanner (MAGNETOM Allegra, Siemens, Erlangen, Germany) equipped with echo planar imaging (EPI) capability and a standard transmit/receive head coil. Images were acquired using a standard gradient-echo EPI sequence: 36 slices; TR = 3000 ms for patients and TR = 2600 ms for controls; TE = 30 ms; matrix size 64 × 64, FOV = 192 × 192; slice thickness 3 mm; axial slice orientation. A T1-weighted anatomical image was also acquired (160 slices; TR = 2300 ms; TE = 3 ms; FOV = 192 × 192; scanning matrix 256 × 256; slice thickness 1 mm; sagittal slice orientation).

The stimulation electrodes were connected with a custom-made, high frequency shielded cable to a battery-powered nerve stimulator (Micromed, Treviso, Italy) located outside the magnet room. The cathode was placed proximal to the anode. The electrical stimulus was a constant-voltage rectangular wave delivered at a rate of 3 Hz with pulse duration of 0.1 ms. To locate the median-nerve at the wrist, the stimulation intensity and electrode sites were modified until stimulation produced an observable thumb twitch. The necessary current for reaching the motor threshold varied between subjects (range 9–15 mA) and patients (range 8–24 mA); it was kept constant along the experiment. Current stimulation during fMRI recording was done in an alternating sequence. In a block design paradigm, a 26-s period with no stimulus was followed by a 26-s period of stimulation. A total of 130 volumes for patients and 110 volumes for subjects were acquired by alternating six or five blocks of stimulation and seven or six blocks in resting state conditions, respectively. In each patient, stimulation of both the right and left median nerve was performed; in healthy volunteers only the right side was stimulated. In order to compare patients and subjects results also for the left stimulation, the fMRI data of controls with right median-nerve stimulation were reversed left to right.

### Image analysis

Data analysis was performed by using the FSL software package (FMRIB Software Library, Oxford University, UK; http://fsl.fmrib.ox.ac.uk/fsl/fslwiki/). Preprocessing of fMRI data included the motion correction (MCFLIRT), slice-time correction, linear trend removal by temporal high-pass filtering (100 s), and spatial smoothing using an isotropic Gaussian filter with a full-width-at-half-maximum of 6 mm^3^. FLIRT was used to register brain structural images to functional ones and transform them into the Talairach coordinate space. Activated voxels were identified with a single-subject general linear model (GLM) for time series data^32^. To account for the hemodynamic delay, the boxcar waveform representing the rest and task conditions was convolved with a double-Gamma hemodynamic response function with temporal derivative. To account for any residual effects of subject movement, six motion parameters (three translations and three rotations) were included as confound regressors in the model.

The first level of statistical analysis of the functional data (at the individual control and PME subject level) was carried out using a GLM-based approach. Brain activation was obtained by comparing BOLD signal intensities in the fMRI images acquired during the stimulation task and at rest, respectively. Individual statistical maps were thresholded at *z* > *2.3, p* < *0.01*, FWE-corrected cluster extent threshold *p*_*FWE*_ < *0.05*^33^.

For each fMRI group a fixed-effects group analysis was performed (*Z* > *2.3, p* < *0.01*, FWE-corrected cluster extent threshold *p*_*FWE*_ < *0.05*). The location of the maximum intensity voxel was expressed as *x, y* and *z* standard coordinates (Montreal Neurological Institute, MNI space), the location of the centre of gravity (COG) of the cluster was obtained as the average of the coordinates weighted by the intensities within the cluster, and the volumes of activation were calculated as the number of activated voxels. Brain parcellation was performed using FSL and was based on the Harvard-Oxford Probabilistic MRI Atlas. This involved extracting 96 cortical regions (48 per hemisphere), 16 subcortical regions (i.e. thalamus, caudate, putamen, pallidum, amygdala, nucleus accumbens, hippocampus, and brain stem, both in the right and left hemisphere) and 27 cerebellar regions.

Due to the low number of subjects involved in the study, between-group differences (controls vs. patients) were statistically assessed using a non-parametric method^34^, specifically a permutation-based inference. This test was carried out with the “randomise” function of FSL by using the beta (COPE) estimates from each subject and patient in MNI space with 5,000 permutations. The significance threshold was set at *p* < *0.01*, FWE-corrected cluster extent threshold *p*_*FWE*_ < *0.05*.

## Additional Information

**How to cite this article:** Storti, S. F. *et al*. Neurophysiological and BOLD signal uncoupling of giant somatosensory evoked potentials in progressive myoclonic epilepsy: a case-series study. *Sci. Rep.* **7**, 44664; doi: 10.1038/srep44664 (2017).

**Publisher's note:** Springer Nature remains neutral with regard to jurisdictional claims in published maps and institutional affiliations.

## Figures and Tables

**Table 1:** Clinical profiles of patients.

	P1	P2	P3	P4
**Age at onset**	9	16	9	6
**Age at which the MRI was performed**	28	52	28	42
**Seizure frequency**	−	−	1 tonic-clonic seizure/month	−
**Myoclonus**	mild	moderate	severe	severe
**Ataxia**	−	−	++	++
**Other symptoms**	−	depression	−	deafness, personality disorder
**Treatment**	VPA, ZNS	VPA, CZP, ZNS, TPM	VPA, ZSN, CZP	VPA, LEV, LTG, CZP
**EEG background**	normal	slow (theta)	normal	slow (theta)
**EEG discharges**	++	−	+	++
	diffuse SW	−	diffuse SW	frontocentral and posterior S

P1, P3: female; P2, P4: male; (++) myoclonic jerks on waking, more than one episode per month; (+) myoclonic jerks on waking, less than one episode per month; S: spike; SW: spike–wave; VPA: valproic acid; ZNS: zonisamide; CZP: clonazepam; TPM: topiramate; LEV: levetiracetam.

**Table 2:** Group analyses of BOLD cortical activation after left and right median-nerve stimulation at 3 Hz in myoclonic patients.

	ROI	Voxels	MAX	MAX X (mm)	MAX Y (mm)	MAX Z (mm)	COG X (mm)	COG Y (mm)	COG Z (mm)
RS	PRG.L/POG.L	4929	4.99	−30	−26	53	−34.5	−21.9	52.8
LS	PO.R/H.R/INS.R	18540	7.36	55	−20	9	51.3	−16.3	6.04
	PRG.R/POG.R	15982	14	37	−18	53	35.2	−24.3	55.9
	CO.L/POG.L/INS.L	10689	5.74	−65	−23	21	−55.2	−21.9	21.4
	CER.V.L	6480	7.27	−22	−48	−17	−13.5	−50.8	−14.2

RS = right stimulation; LS = left stimulation; Voxels = the number of voxels in the cluster; MAX = the value of the maximum z-statistic within the cluster; MAX X/Y/Z (vox/mm) = the location of the maximum intensity voxel, given as X/Y/Z coordinate values in standard space coordinates (mm); COG X/Y/Z (vox/mm) = the location of the centre of gravity for the cluster (a weighted average of the coordinates by the intensities within the cluster); PRG = precentral gyrus; POG = postcentral gyrus; PO = parietal operculum cortex; H = Heschls gyrus (includes H1 and H2); INS = insular cortex; CO = central opercular cortex; CER = cerebellum; L = left; R = right.

**Table 3:** Statistical comparisons between controls and patients after left and right median-nerve stimulation at 3 Hz.

		Controls vs. Patients								
		ROI	Voxels	MAX (1-p)	MAX X (mm)	MAX Y (mm)	MAX Z (mm)	COG X (mm)	COG Y (mm)	COG Z (mm)
RS	Cts > Pts	SGp.R	12524	0.671	68	−35	13	44.9	−40.5	41.7
		POG.L	8742	0.643	−45	−24	29	−43.4	−27.7	47.2
		Thal.L	2121	0.4	−15	−19	−10	−17.1	−22.3	−1.91
		PT.L	2117	0.4	−67	−18	0	−56.4	−39.4	19.8
		F3o.R	1887	0.371	51	22	19	46.7	18.2	27
		PRG.R	1584	0.329	48	11	9	54.5	7.56	18.8
		FP	1346	0.286	36	46	−8	37	46.4	2.17
		TO2.L	1335	0.286	−63	−48	0	−61.7	−55.5	7.19
		T3p.L	1289	0.286	−53	−41	−28	−48.5	−30.3	−15.4
LS	Cts > Pts	FP.L	3608	0.486	−31	42	−19	−36.1	45.7	−4.16
		SPL.L	3077	0.471	−35	−51	31	−31.5	−51	46.3
		SPL.R	2580	0.414	24	−49	42	18.8	−54.7	51.3
		CO.R	2508	0.414	51	−26	15	36.3	−21.4	27
		CER.VIIb.R	1638	0.3	16	−71	−51	22.3	−68.6	−43.3
		CER.CrusI.L	1474	0.3	−53	−65	−34	−42.9	−72.5	−23.9
		F3o.L	1083	0.257	−34	16	15	−43.4	20.2	22.1
	Pts > Cts	Accbns.L.R/Put.L.R	5381	0.6	22	4	−48	3.41	5.32	−15.6
		T3p.R	1434	0.286	62	−31	−19	56.9	−33.5	−10.3
		OLs.L	1316	0.257	−49	−69	20	−46.5	−71.4	36.1
		TP.L	1139	0.257	−29	4	−35	−20.1	4.61	−27.8
		TFa.L	1014	0.243	−28	−1	−50	−34.6	−1.83	−41

RS = right stimulation; LS = left stimulation; Cts > Pts = controls > patients; Pts > Cts = patients > controls. Of note that during right stimulation for patients > controls no cluster activity was detected. The minimum cluster size reported is 1000 voxels. Voxels = the number of voxels in the cluster; MAX = the value of the maximum z-statistic within the cluster; MAX X/Y/Z (vox/mm) = the location of the maximum intensity voxel, given as X/Y/Z coordinate values in standard space coordinates (mm); COG X/Y/Z (vox/mm) = the location of the centre of gravity for the cluster (a weighted average of the coordinates by the intensities within the cluster); SGp = supramarginal gyrus, posterior division; POG = postcentral gyrus; Thal = Thalamus; PT = planum temporale; F3o = inferior frontal gyrus, pars opercularis; PRG = precentral gyrus; FP = frontal pole; TO2 = middle temporal gyrus, temporooccipital part; T3p = inferior temporal gyrus, posterior division; SPL = superior parietal lobule; CO = central opercular cortex; CER = cerebellum; Accbns = accumbens; Put = putamen; OLs = Lateral Occipital Cortex, superior division; TP = temporal pole; TFa = Temporal Fusiform Cortex, anterior division; L = left; R = right.
